# 28. Utilizing the Charleston Comorbidity Index as an Independent Predictor for Outcomes in SARS-Cov-2 Positive Patients

**DOI:** 10.1093/ofid/ofab466.028

**Published:** 2021-12-04

**Authors:** Irene Riestra Guiance, Steven Char, Ernesto Robalino Gonzaga, Isabel Riestra, Minh Q Ho

**Affiliations:** 1 UCF/HCA GME Consortium, Kssimmee, Florida; 2 UCF/HCA Healthcare GME, Weehawken, New Jersey; 3 Suffolk University, Maitland, Florida; 4 Orlando VA Healthcare System, 14014 Deep Forest Court, Florida

## Abstract

**Background:**

Since COVID-19 was declared a pandemic, it has seemed that the virus is nondiscriminatory causing 3.73 million deaths worldwide. The Charleston Comorbidity Index (CCI) is a scoring system predicting the one-year mortality for patients with a range of comorbid conditions and is widely used as a predictor of prognosis and survival for a range of pathologies. This study aims to assess if there is an impact of comorbidity burden on COVID-19 patients by utilizing their CCI score.

Charleston Comorbidity Index Score

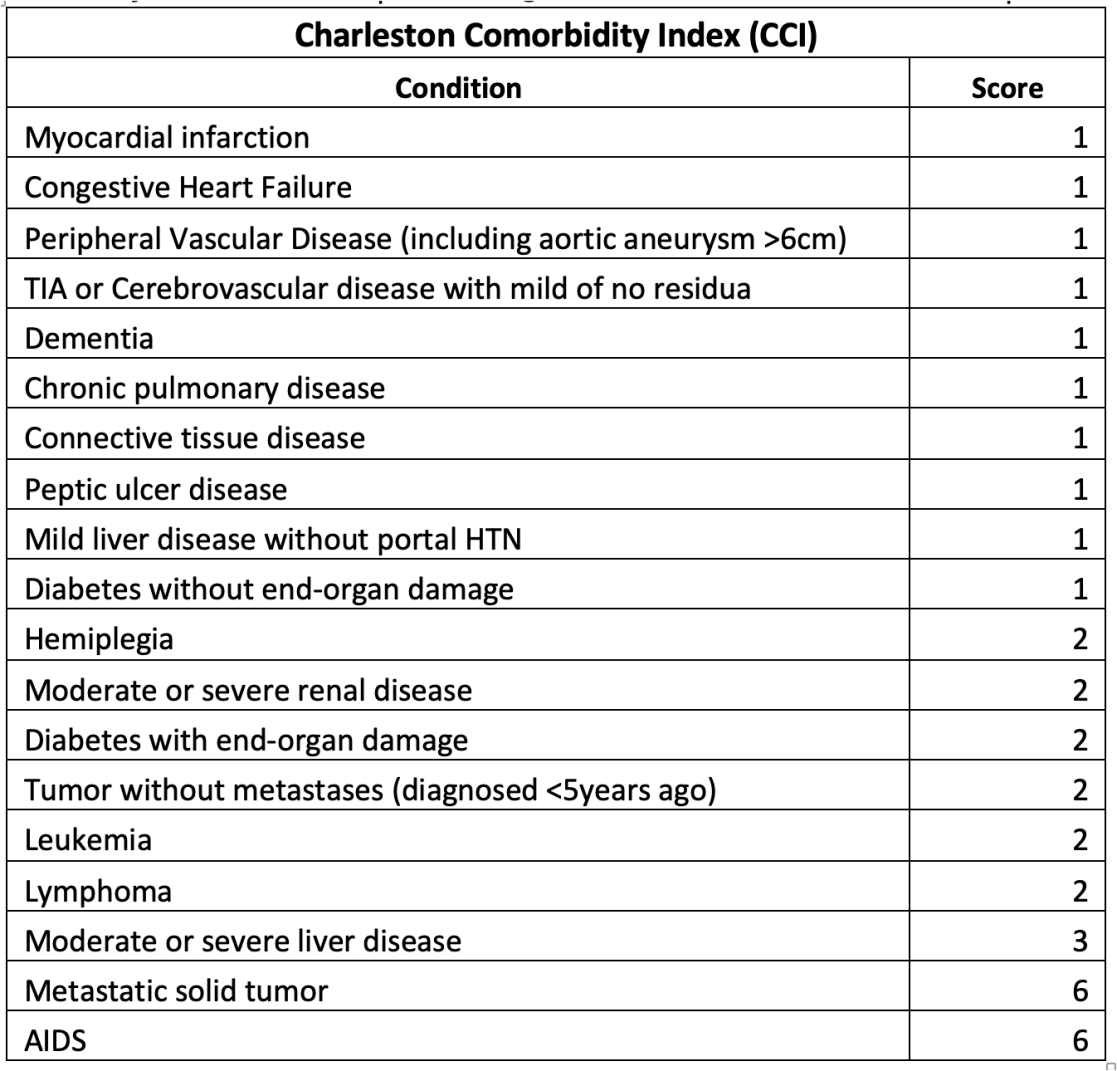

Scoring system for Charleston Comorbidity Index (CCI). Plus 1 point for every decade age 50 years and over, maximum 4 points. Higher scores indicate a more severe condition and consequently, a worse prognosis.

**Methods:**

Multicenter, retrospective review of patients diagnosed with COVID-19 from January 2020 to September 2020 throughout the HCA Healthcare system. CCI scores for all COVID-19 positive patients were calculated and logistic regression analysis was performed to predict hospitalization and ICU admission by CCI controlling for age, sex and race. A multinomial regression model was also performed to predict discharge status by CCI controlling for age, sex and race. ROC curves to indicate the CCI cut-off point for each outcome (hospitalization, ICU admission and mortality) was performed, and Youden’s Index was used to identify the optimal point.

**Results:**

In the study timeframe, 92,800 patients were diagnosed with COVID-19 and of those, 48,270 were hospitalized. A one-point increase in CCI was associated with higher odds of hospitalization [OR 1.718; 95% CI 1.696-1.74]. The threshold for significance to predict hospitalization was a CCI of 1.5 (AUC 0.804, Youden Index 0.48) with a specificity (73%) and sensitivity (75%). A one-point increase in CCI was associated with 1.444 higher odds of an ICU admission (95% CI 1.134-1.155). A one-point increase in CCI significantly increased the odds of discharge to hospice compared to any discharge other than hospice [OR 1.162; 95% CI 1.142-1.182]). A one-point increase in CCI score was associated with 1.188 higher odds of in-hospital mortality (95% CI, 1.173-1.203) with a CCI threshold of 3.5 having the highest specificity (50.9%) and sensitivity (79.9%) to predict mortality outcome (AUC 0.704, Youden Index 0.31).

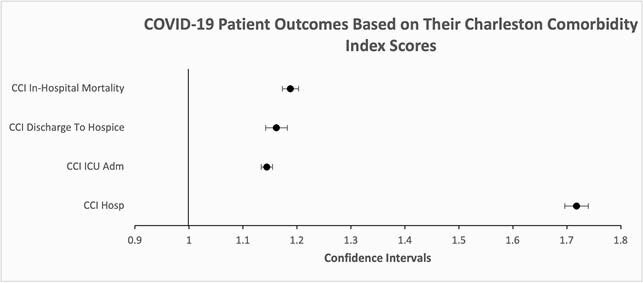

**Conclusion:**

In conclusion CCI score is an adequate predictor of hospitalization and in-hospital mortality but less so in predicting ICU admission in COVID-19 positive patients.

**Disclosures:**

**All Authors**: No reported disclosures

